# The habitat quality paradox: loss of riparian forest cover leads to decreased risk of parasitism and improved body condition in an imperiled amphibian

**DOI:** 10.1093/conphys/coad101

**Published:** 2024-01-28

**Authors:** Catherine M Bodinof Jachowski, Valentina Alaasam, Arden Blumenthal, Andrew K Davis, William A Hopkins

**Affiliations:** Department of Forestry and Environmental Conservation, Clemson University, Clemson, SC 29634, USA; Department of Fish and Wildlife Conservation, Virginia Tech, Blacksburg, VA 24061, USA; Department of Biology, New York University, New York, NY 89503, USA; Department of Fish and Wildlife Conservation, Virginia Tech, Blacksburg, VA 24061, USA; Odum School of Ecology, University of Georgia, Athens, GA 30602, USA; Department of Fish and Wildlife Conservation, Virginia Tech, Blacksburg, VA 24061, USA

**Keywords:** body condition, condition indices, Cryptobranchus alleganiensis, forest cover, Hellbender, hematology, land use, parasitism, physiology

## Abstract

Amphibian declines are a global phenomenon but responses of populations to specific threats are often context dependent and mediated by individual physiological condition. Habitat degradation due to reduced riparian forest cover and parasitism are two threats facing the hellbender salamander (*Cryptobranchus alleganiensis*), but their potential to interact in nature remains largely unexplored. We investigated associations between forest cover, parasitic infection and physiology of hellbenders to test the hypotheses that physiological condition responds to infection and/or habitat degradation. We sampled 17 stream reaches in southwest Virginia, USA, on a year-round basis from 2013 to 2016 and recorded 841 captures of 405 unique hellbenders. At each capture we documented prevalence of two blood-associated parasites (a leech and trypanosome) and quantified up to three physiological condition indices (body condition, hematocrit, white blood cell [WBC] differentials). We used generalized linear mixed models to describe spatiotemporal variation in parasitic infection and each condition index. In general, living in the most heavily forested stream reaches, where hellbender density was highest, was associated with the greatest risk of parasitism, elevated neutrophil-to-lymphocyte (N:L) ratios and eosinophils, slightly lower hematocrit and lower mean body condition in hellbenders. All condition indices fluctuated temporally in a manner consistent with seasonal variation in hellbender metabolic demands and breeding phenology and were associated with land use during at least part of the year. Paradoxically, relatively low levels of forest cover appeared to confer a potential advantage to individuals in the form of release from parasites and improved body condition. Despite improved body condition, individuals from less forested areas failed to exhibit fluctuating body condition in response to spawning, which was typical in hellbenders from more forested habitats. We postulate this lack of fluctuation could be due to reduced conspecific competition or reproductive investment and/or high rates of filial cannibalism in response to declining forest cover.

## Teaser

We sampled imperiled hellbender salamanders (*Cryptobranchus alleganiensis*) along a land use gradient to investigate drivers of physiological condition. Paradoxically, living in the highest quality habitat was associated with the greatest risk of parasitism, elevated neutrophil-to-lymphocyte (N:L) ratios and eosinophils, slightly lower hematocrit, and lower mean body condition in hellbinders.

## Introduction

Amphibians currently rank among the most endangered vertebrates on Earth, with 40.7% of species currently listed as either vulnerable, endangered or critically endangered by the International Union for Conservation of Nature (Luedtke *et al.*, 2023). While amphibian population declines are considered a global phenomenon resulting from broad-scale patterns of habitat loss, disease, pollution, climate change and introduced species (Collins and Storfer, 2003; Hof *et al.*, 2011; Green *et al.*, 2020), responses to specific threats can be highly variable among species and between sub-populations of the same species (Grant *et al.,* 2020). For example, emerging infectious disease and associated declines of many amphibians have been attributed to a variety of parasites and pathogens (Daszak *et al.*, 1999; Yap *et al.*, 2015; Fisher and Garner, 2020). However, because exposure to a pathogen does not always result in clinical disease or altered demographic rates, disease-driven declines are increasingly viewed as a complex and context-dependent response involving attributes of host, parasite/pathogen and the environment (Carey *et al.*, 1999; Cohen *et al.*, 2019). Identifying situations where a threat is most likely to elicit deleterious individual- and population-level outcomes can aid conservation by highlighting regions, species or population segments at greatest risk and thus in greatest need of protection.

Habitat degradation driven by land use alteration is one of the most ubiquitous threats facing amphibians (Gallant *et al.*, 2007; Grant *et al., *2016; 2020) and may define the context that determines how pathogen and parasite exposure manifests in a population (Sutherst, 2001; Hayes *et al.*, 2010). Development of land for residential, urban and agricultural use can influence both abiotic and biotic attributes of freshwater habitats (Allan, 2004) and thereby influence disease dynamics in various ways. For example, land use can influence parasite occurrence on the landscape (Koprivnikar *et al.,* 2012; Koprivnikar and Redfern, 2012) by defining aquatic habitat connectivity, local host density and thus rates of transmission among amphibian hosts (McKenzie, 2007), particularly when parasites are reliant on density-dependent transmission (Lafferty and Kuris, 1999; Schotthoefer *et al.*, 2011; Rohr *et al.*, 2015). Additionally, because conversion of land for anthropogenic use can degrade the quality or abundance of aquatic resources amphibians depend on, it may lead to increased physiological demands on hosts and potentially increase susceptibility to otherwise benign parasites (Koprivnikar, 2010; Blaustein *et al.*, 2012; Romero and Wingfield, 2015). Though experimental work has advanced our understanding of how parasites and pathogens might interact with land use (or associated pollutant exposure) to affect amphibian physiology, growth and survival *ex situ* (Koprivnikar *et al.*, 2012), few studies have attempted to describe associations between land use, parasite distributions and host physiology in wild amphibian populations (Marcogliese *et al.*, 2009).

The goal of our study was to investigate how land use might influence host–parasite dynamics and physiological condition of a critically imperiled amphibian. Our focal species was the eastern hellbender, *Cryptobranchus alleganiensis alleganiensis*, which is a large, long-lived, fully aquatic, stream-dwelling salamander of great conservation concern (USFWS, 2019). In our study system, we find that loss of riparian forest cover is associated with declines in adult population density, probability of occurrence, reproductive success and recruitment of hellbenders, and that degraded water quality likely functions as a mechanism linking forest cover to population level responses (Bodinof Jachowski *et al.*, 2016; Bodinof Jachowski and Hopkins, 2018; Hopkins *et al.*, 2023). In particular, specific conductance (one measure of salinity) exhibits a strong negative correlation with riparian forest cover in our system (see Supplementary Figure S1). Because elevated conductivity often serves as a non-specific indicator of waterborne pollutants and water quality impairment (Allan and Castillo, 2007), we consider riparian forest cover to function as a proxy of in-stream hellbender habitat quality in our system, such that habitat quality increases as riparian forest cover increases. Notably however, the associations between riparian forest cover and hellbender parasitic infection or between riparian forest cover and hellbender physiological condition in our system have not been explored.

**Figure 1:**
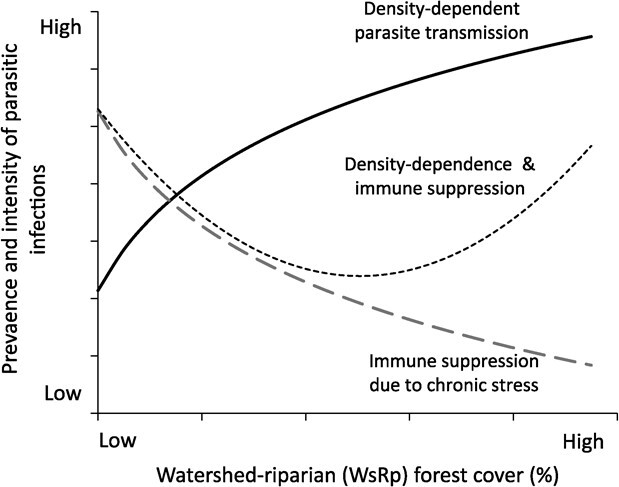
Alternative predicted associations between prevalence and intensity of parasitic infections and riparian forest cover within the contributing watershed under the hypothesis that forest cover influences parasite dynamics indirectly, through alterations in host density and thus opportunities for parasite transmission (i.e. host density-dependent parasite transmission) and/or alterations in host vulnerability due to variation in habitat quality (i.e. host immune suppression due to chronic physiological stress; Romero and Wingfield, 2015).

Two potentially important parasites of hellbenders in our system include a leech (*Placobdella appalachiensis*, Hopkins *et al.*, 2014) and an unknown species of trypanosome blood parasite (Davis and Hopkins, 2013). Unpublished data collected by one of us (W.A. Hopkins) indicates leeches can function as a vector for trypanosome transmission among hellbenders in *ex situ* experimental conditions, though it is unknown whether leeches are the only vector of the blood parasite *in situ*. While the effects of the trypanosome on hellbenders remain unclear (Hopkins *et al.*, 2016), *P. appalachiensis* can disrupt the normal adrenocortical response (i.e. increase in plasma glucocorticoids in response to an acute stressor) of their host (DuRant *et al.*, 2015) and provoke an immune response (Hopkins *et al.*, 2016). This is important because the adrenocortical response is generally considered to be an adaptive response that mediates the mobilization of energy stores in response to acute disturbance (Wingfield and Boonstra*,* 2013). Prior to the current study, both parasites had only been documented in hellbenders occupying a narrow land use gradient (69–70% forest throughout the upstream riparian corridor) spanning approximately 17 km of a single stream in southwest Virginia, USA, though little effort had been made to screen for either parasite in hellbenders throughout the broader region.

In our study we focused on answering two general questions concerning effects of land use and parasites on hellbenders. Our first question was whether land use influences host–parasite dynamics in our system. For this question, we hypothesized that forest cover influences parasite prevalence and intensity indirectly, through alterations in host density and thus opportunities for parasite transmission (i.e. density-dependent parasite transmission) and/or alterations in host vulnerability to infection due to variation in habitat quality (i.e. immune suppression due to chronic physiological stress; Romero and Wingfield, 2015; Davis *et al.*, 2008). To answer our question, we sampled hellbenders across a land use gradient on a year-round basis and screened individuals for leeches and trypanosomes. Under our hypothesis we predicted three possible outcomes, including that parasite prevalence and/or intensity would either be 1) highest in the most heavily forested, and thus highest quality, habitats (i.e. host density-dependence as a primary driver), 2) highest in the least forested reaches (i.e. host immune suppression as primary driver) or 3) highest at both upper and lower extremes of forest cover (i.e. density dependence and immune suppression both acting as drivers; Fig. 1).

Our second question was whether hellbender physiological condition is responsive to land use, parasitism or both. Here we hypothesized hellbender physiological condition would respond to both forest cover, give that it is a proxy of habitat quality (Supplementary Figure S1; Bodinof Jachowski and Hopkins, 2018) and parasitic infection (Bower *et al.*, 2019). To evaluate support for our hypothesis we used three complementary metrics to describe hellbender physiological condition, including two blood parameters (hematocrit and white blood cell profiles) and a body condition index. We predicted that hellbenders would exhibit an increase in hematocrit in response to declining riparian forest cover, a decrease in hematocrit in response to parasitism, an increase in neutrophil-to-lymphocyte ratios in response to both stressors and a decrease in body condition index in response to both stressors.

## Materials and Methods

### Species Background

Hellbenders are large (up to 74 cm), long-lived (25+ years), fully aquatic amphibians native to much of the eastern U.S. (Taber *et al.*, 1975). They depend on rock crevices for shelter and nesting and rely primarily on cutaneous respiration and thus well-oxygenated water (Guimond and Hutchison, 1973). Typical habitat includes swift flowing, cool, rocky streams, but the species is occasionally found in large rivers with water temperatures that exceed 25°C in summer. Sub-adults and adults consume crayfish as their primary prey item and exhibit high site fidelity to specific stream reaches and cavities beneath boulders and bedrock (Nickerson and Mays, 1973*a*-b; Bodinof *et al.*, 2012*a*). Spawning occurs annually during a brief period (Aug 21-Sep 20 in southwest VA) when adults aggressively compete for access to nest sites and females deposit several hundred eggs in a nest cavity guarded by a single male (Hopkins *et al.*, 2023). Hatching occurs at 60 to 90 days post oviposition and males remain with hatchlings until larval emergence in early spring (April–May); though both sexes are known to occasionally cannibalize eggs (Hopkins *et al.*, 2023).

### Study Area

Our study area included the New River and South Fork Holston River drainages in southwestern Virginia, which are described in detail by Bodinof Jachowski *et al.* (2016). In total we sampled hellbenders in 17 stream reaches from seven streams (n = 1–7 reaches per stream) representing a gradient in watershed riparian (WsRp) forest cover (Table 1). Briefly, the WsRp area is a pre-defined spatial extent summarized in the StreamCat (Hill *et al.*, 2016) database where *watershed* refers to all land within the upstream contributing area for a stream reach and r*iparian area* refers to all land within a 100 m buffer of a stream (NHDPlus v2 dataset; McKay *et al.*, 2019). As such, characteristics of the WsRp area reflect the riparian conditions over a broad spatial scale upstream of a stream reach. We quantified the percent of pixels (30 m resolution) classified as forest cover (evergreen, deciduous or mixed forest) within the WsRp area for each stream reach according to the 2016 National Land Cover Dataset (Dewitz, 2019). Notably, percent WsRp forest cover was inversely correlated with agriculture (*r* = −0.90), the latter of which was the most widespread form of anthropogenic land use alteration in our study area (Bodinof Jachowski *et al.*, 2016). Our previous work shows that specific conductance is negatively correlated with WsRp forest (see Supplementary Figure S1; Bodinof Jachowski and Hopkins, 2018). As such, relied on WsRp forest as a proxy of in-stream habitat quality and use WsRp forest and habitat quality interchangeably hereafter.

**Table 1:** Summary of hellbender (*Cryptobranchus alleganiensis*) captures from 17 stream reaches in southwest Virginia, USA used to investigate the association between forest cover, parasitic infection, body condition quantified as a scaled mass index (SMI), hematocrit (HMC) and white blood cell differentials (WBC)

Reach	River	Order	WsRp Forest (%)	Infection Status				Response Variables						
				None	Tryp -only	Leech only	Co-infection	Parasite Occurrence	Leech Prevalence	Leech Intensity	Tryp Prevalence	SMI	HMC	WBC
1	1	5	54.5	8				8	0	0	0	8		8
2	2	4	57.1	6				6	0	0	0	6	3	6
3	3	4	57.2	4				4	0	0	0	4		4
4	4	6	57.3	1				1	0	0	0	1	1	
5*	3	4	57.5	51				51	0	0	0	51	37	22
5	3	4	59.0	1				1	0	0	0	1	1	1
7	4	5	61.1	1				1	0	0	0	1		
8*	5	4	61.5	26				26	0	0	0	26	20	13
9	4	5	61.5	1				1	0	0	0	1		
10	5	3	62.7	11				11	0	0	0	11	2	9
11*	5	4	64.7	26	162	20	123	331	331	143	331	321	192	104
12	6	4	66.3	1				1	0	0	0	1		
13	5	3	66.3	8				8	0	0	0	8	4	3
14*	5	4	66.6	26	125	18	107	276	276	125	276	266	182	100
15*	5	3	66.8	35	6	2	1	44	44	3	44	41	28	17
16	5	3	67.6	5				5	0	0	0	5		5
17*	7	3	83.2	42	24			66	0	0	66	62	18	27
Total				253	317	40	231	841	651	271	717	814	488	319

Reach: a unique identifier for stream reach; Order: refers to stream order, which is a measure of reach position within the watershed; WsRp: watershed-riparian; Tryp: trypanosome; * Indicates reaches intensively surveyed as part of a separate study (Bodinof Jachowski and Hopkins, 2018) to investigate hellbender demography.

### Physiological Condition Indices

We considered body condition, hematocrit and white blood cell profiles as three complementary measures of hellbender physiological condition.

#### Body condition

Body condition refers to the state of an individual’s energetic stores, where individuals harboring greater energy reserves to devote to growth and maintenance are often regarded as having an advantage (Labocha *et al.*, 2014; but see Wilder *et al.*, 2016). Indices used to quantify body condition are typically calculated as a measure of relative mass given an individual’s structural size (Jakob *et al.*, 1996; Green, 2001; Peig and Green, 2009). Among amphibians, body condition has been associated with dispersal probabilities (Lowe *et al.*, 2006), survival and reproductive success (Semlitsch, 1987; Reading and Clarke, 1995; Reading, 2007; Scott *et al.*, 2007; Garner *et al.*, 2011; Bodinof *et al.*, 2012*b*). Body condition can vary due to resource availability and acquisition or an individual’s ability to cope with environmental alterations, like those that often accompany habitat loss (Jakob *et al.*, 1996). Directional responses of body condition to parasitism are known to be highly variable among species and negative effects of parasites on body condition are often less pronounced for ectotherms relative to endotherms (Sánchez *et al.*, 2018). However, because body condition can fluctuate rapidly in response to spawning and feeding in ectotherms (Brown and Murphy, 2004), including hellbenders (Hopkins *et al.*, 2023), we considered it plausible that hellbender body condition profiles might signal biologically relevant responses to land use or parasites.

#### Hematocrit

Hematocrit is the percentage of whole blood volume composed of red blood cells (Hillman, 1976). Hematocrit can vary seasonally and is positively associated with blood oxygen carrying capacity in vertebrates (Harris, 1972). We chose to focus on hematocrit in hellbenders because both parasites in our system are strongly affiliated with blood and because hematocrit levels can be indicative of anemia, dehydration and parasitic infection in other systems (Toque, 1993). While effects of blood parasites like *Trypanosoma* on amphibian hosts is poorly studied in general (Arikan and Cicek, 2014; Forzán *et al.*, 2017), trypanosomiasis can lead to destruction of red blood cells and anemia in non-amphibian hosts (Stijlemans *et al.*, 2018; Kipkorir *et al.*, 2021). Additionally, hematocrit can respond to changes in temperature (Biron and Benfey, 1994; Roche and Bogé, 1996) and exposure to water pollutants (Al-Attar, 2005) which often accompany loss of forest cover.

#### White blood cell profiles

We describe white blood cell (WBC) profiles using differential WBC counts. Briefly, WBC differentials refer to relative proportion of circulating white blood cells made up of each major white blood cell type and are a commonly used indicator of health for a wide range of vertebrates including amphibians (Davis *et al.*, 2008). Importantly, WBC profiles fluctuate with season and life stage and thus must be considered in context for accurate interpretation. For example, exposure to cool temperatures suppresses lymphocyte production in amphibians, resulting in a gradual increase in the ratio of neutrophils to lymphocytes (i.e. hereafter N:L ratios) during transitions from warm to cool seasons (Maniero and Carey, 1997; Raffel *et al.*, 2006; Barriga-Vallejo *et al.*, 2015). However, due to differences in function each cell type, proliferation (or lack thereof) of one or more type within a given season can provide some insights into the type of stressor an organism may be experiencing (Davis *et al.*, 2008). Because proliferation of neutrophils, a major phagocytic WBC, is one of the first responses to infection, inflammation and stress (Allender and Fry, 2008; Davis *et al.*, 2008), elevated N:L ratios are often indicative of an active innate immune response or a relative increase in circulating glucocorticoid hormones (i.e. stress hormones) (Davis *et al.*, 2008; Davis and Maerz, 2022). Additionally, proliferation of eosinophils is often indicative of parasitic infection, elevated monocytes are indicative of inflammation and defense against infection (including bacterial), and exposure to environmental pollutants, such as pesticides, has been linked to decreasing lymphocytes (Christin *et al.*, 2003).

### Field Sampling

We sampled hellbenders on a year-round basis between 2013 and 2016 (Supplementary Figure S2). All surveys occurred between 0825 and 1748 h. For study reaches that we visited multiple times, sequential visits were separated by at least 14 days. To locate and capture hellbenders, we used snorkeling while turning rocks (Nickerson *et al.*, 2003), reaching under boulders to search by tactile means and visual surveys of artificial shelters deployed as part of concurrent studies (Bodinof Jachowski *et al.*, 2016; Bodinof Jachowski and Hopkins, 2018; Bodinof Jachowski *et al.*, 2020). Upon capture we quickly transported hellbenders to the bank for processing. We marked individuals with unique coded passive integrated transponder (PIT) tags (models HPT8 or HPT12; Biomark Inc., Boise, ID, USA) which we inserted subcutaneously along the dorsolateral base of the tail. We recorded sex based on external morphology (cloacal swelling in males) when evident, weighed each individual to the nearest 5 to 10 g using Pesola® spring scales (Pesola AG, Schindellegi, Switzerland) and recorded total length to the nearest cm. We conducted one to three visual scans to determine the presence of ectoparasites and recorded the total number of leeches when they were detected. We determined the presence of trypanosomes by screening blood samples in the lab (see Supplemental Information).

#### Blood collection and processing

We obtained whole blood samples (≥ 50 μL; but not more than 100 μL/100 g body mass) for measuring hematocrit, quantifying WBC differentials and trypanosome screening. Blood samples were typically collected within three minutes of making initial physical contact with a hellbender (hereafter, ‘capture’) (median time to blood collection = 2.33 ± 12.79 minutes, *n* = 832), but on some occasions were collected 30 to 139 minutes post-capture (n = 49 of 832 samples). Because acute changes in hematocrit have been associated with handling (Biron and Benfey, 1994) and we were interested in baseline conditions, we only considered samples collected within three minutes of capture when statistically analyzing hematocrit. Because DuRant *et al.* (2015) demonstrated a lag time of 6+ hours for hellbender WBC profiles to respond to handling we included all available samples in our WBC statistical analysis, regardless of time to blood collection. We calculated body condition as a scaled mass index (SMI), where scaled mass refers to the estimated mass of an individual if it were of a reference structural size (Peig and Green, 2009; MacCracken and Stebbings, 2012). A full description of methods for preparation of blood smears, trypanosome screening and quantification of hematocrit, WBC differentials and body condition are provided elsewhere (see Supplemental Information).

### Data Analysis

In all our analyses, we restricted our focus to sexually mature adults (≥ 29 cm) for which leech infection status, trypanosome infection status, total length and body mass were known. When assessing patterns of parasite occurrence we included all individuals (males, females and unknown sex individuals) because we wanted to maximize our ability to detect parasites in a stream reach if they were present (i.e. minimize false negatives). However, when assessing physiological endpoints (SMI, hematocrit and WBC) we limited our focus to adults of known sex for two reasons. First, inclusion of unknown sexes in our analyses of hellbender condition endpoints unnecessarily complicated model structure, as it required a three-level term to account for sex effects (i.e. male, female, unknown) and the small number of ‘unknown sex’ samples (see Results) precluded our ability to estimate meaningful effect sizes for the unknown sex level. Second, we found (*post-hoc*) that including or excluding unknown sex individuals had no influence on our results or final inferences.

Prior to analysis, we used Pearson’s correlation coefficients to screen for correlation among physiological indices used as endpoints, where we considered correlations problematic (i.e. indicative of non-independence) when |r| ≥ 0.7. Scaled mass index was not correlated with hematocrit (r = 0.03), % neutrophils (r = 0.04), % lymphocytes (r = −0.01), % eosinophils (r = −0.07) or N:L ratios (r = 0.04). Hematocrit was only moderately correlated with % neutrophils (r = −0.40), % lymphocytes (r = 0.43), % eosinophils (r = −0.36) and N:L ratios (r = −0.42). Because WBC parameters were calculated as (or from) proportions we considered them all to be interdependent. To account for interdependence and reduce dimensionality we performed a principal components analysis (PCA) based on a correlation matrix of four WBC endpoints (N:L ratios, % neutrophils, % eosinophils and % lymphocytes) prior to further analysis. Before performing the PCA we transformed each endpoint to meet assumptions of normality ($\sqrt{\% eosinophils}$, $\sqrt{\% neutrophils}$, $\sqrt{constant-\% lymphocytes}$, $\sqrt[3]{N:L\ ratio}$).

*Effects of land use on host–parasite dynamics—*To understand how land use may have influenced hellbender-parasite dynamics, we investigated the association between forest cover and parasitic infection at two hierarchical scales. In our coarse, landscape-scale, analysis we used conditional density plots to investigate patterns of parasite occurrence among stream reaches. Briefly, conditional density plots describe how the conditional distribution of a factor changes over a continuous independent variable (Venables and Ripley, 2013). To generate conditional density plots we first assigned the state of each sampling reach (*n* = 17) as either occupied or unoccupied, separately for leeches and trypanosomes, based on all hellbenders screened for parasites during the study (see Table 1). We created a plot for each parasite using the graphics package in program R (https://www.rdocumentation.org/packages/graphics/versions/3.6.2/topics/cdplot*accessed* 19 June 2023), where we defined WsRp forest cover as our independent continuous variable and stream reach state (occupied or unoccupied) as our response.

In a finer-scale analysis, we subset our data to include only capture events from stream reaches where parasites were detected and used hurdle models (Feng, 2021) in an information theoretic framework to investigate variation in parasitic infection while treating hellbender capture events as the sampling unit. Briefly, hurdle models were developed to accommodate zero inflated data like those commonly encountered when assessing parasitic infection and involve two separate linear modeling steps. First, we modeled the process giving rise to values of zero in the dataset (i.e. prevalence, defined as the probability of a hellbender being infected), which involved using parasite presence or absence on each capture occasion as our response variable. In this step we used a mixed generalized linear model with a logit link and binomial error distribution (i.e. logistic regression). Next, we subset our data to include only observations from infected individuals and modeled the intensity of infection (defined as abundance of the parasite on/in an infected individual) using a mixed generalized linear model with a log link and a truncated binomial error distribution. We modeled both prevalence and intensity of leech infections. However, we only modeled prevalence of trypanosome infections because we lacked reliable information on trypanosome infection intensity.

Prior to each analysis we screened for evidence of seasonal variation in our response variable. To do so, we first quantified the day of the calendar year (hereafter DOY; 1–365) when each sample was collected and scaled values to have a mean = 0 and sd = 1. Next, we built a series of five screening models to determine the most appropriate form of DOY to use in our main analyses. Our null (intercept-only) screening model assumed no seasonal fluctuation in the response. Our four alternative screening models assumed either a linear (~DOY), quadratic (~DOY + DOY^2^), a non-linear third order polynomial (~DOY + DOY^2^ + DOY^3^) or a non-linear fourth order polynomial (~DOY + DOY^2^ + DOY^3^+ DOY^4^) relationship between DOY and each endpoint. We included a random effect term for hellbender identity (ID) in each screening model to account for repeated sampling of individual hellbenders that were captured on multiple occasions. We used Akaike Information Criterion for small samples (AICc; Burnham and Anderson, 2002) to determine which form of DOY was best supported by our data and used the top-ranking model from the screening procedure as the simplest (base) model in the main analyses for the corresponding endpoint. When seasonal variation was evident, we accounted for it by including the most appropriate form of calendar day of year (DOY; hereafter, season) in all candidate models assessed in our main analysis.

Our base model (~Season) represented the hypothesis that parasite prevalence (or intensity) was best explained by seasonal variation alone. Our first alternative model (~Season + WsRp forest) represented our hypothesis that prevalence/intensity increased with forest cover as a result of positive density-dependence or alternatively, decreased with forest cover as a result of decreased resistance of hellbenders to infection. Our second alternative model (~Season + Reach) represented the hypothesis that patterns of parasitic infection varied among stream reaches due to some attribute other than WsRp forest cover.

#### Effects of land use and parasitism on host physiology

We used mixed generalized linear regression models and an information theoretic approach to investigate factors associated with hellbender physiological condition. In total, we modeled three physiological condition endpoints, including SMI, hematocrit and the first principal component from the ordination performed on WBC parameters (see Results). We used an identity link and Gaussian error distribution when modeling each endpoint, all of which followed a distribution that was approximately normal. As in our analysis of parasitic infection, we screened for evidence of seasonal variation prior to each main analysis and included the best supported form of season in all candidate models when supported.

For each physiological endpoint, we considered six candidate models that included various combinations of season, sex, WsRp forest and parasite infection status (hereafter, infection). Due to the rarity of leech-only infections (Table 1) we defined infection as a factor with three levels (no infection, single infection [leeches or trypanosomes] and coinfection [leeches and trypanosomes]). Prior to model fitting we plotted our data to screen for collinearity between predictors. Notably, WsRp forest cover was confounded with infection (see Results), which precluded our ability to consider additive or interactive effects of WsRp forest and infection. However, because we maintained an interest in understanding which of these factors was the better predictor of hellbender condition, we retained both terms but avoided including them in the same model. Our base model in each analysis (~Season) represented the hypothesis that variation in condition was primarily driven by predictable annual fluctuations in temperature, natural history or resource availability. We used interactions (Season*WsRp forest; Season*Infection; Season*Sex) to represent hypotheses that the effect of season would vary depending on habitat quality, infection status or sex, respectively and used additive forms of each term to represent the hypotheses that effect sizes would be similar throughout the year.

We fit all linear models describing parasitic infection and physiological condition using either the lme4 or GLmmADMB package in Program R (R Core Team, 2013) and, for each analysis, we ranked models according to Akaike Information Criterion adjusted for small samples (AICc; Burnham and Anderson, 2002). We considered models that carried any portion of the upper 90% of AICc model weight to be part of the confidence set of models and warrant further examination. We present the top-ranking model from each analysis in detail but only discuss lower ranking models when they yielded contrasting inference from the top-ranking model. We report mean estimates and their associated variance with 95% confidence intervals based on fixed effects unless otherwise noted.

## Results

We recorded a total of 841 captures of 405 unique adult hellbenders (187 females; 196 males; 22 unknown sex) between 2013 and 2016. Sample sizes varied by season and reach (Supplementary Figure S2) with 94% (794 of 841) of samples collected from six reaches (reaches 5, 8, 11, 14, 15 and 17; Table 1) where concurrent research was occurring to investigate variation in hellbender occurrence and abundance across a land use gradient (Bodinof Jachowski *et al.*, 2016; Bodinof Jachowski and Hopkins, 2018; Bodinof Jachowski *et al.*, 2020).

### Effects of Land Use on Parasite Dynamics

Parasites were only detected in four of 17 reaches and two of the seven streams sampled (Table 1). We detected both trypanosomes and leeches in three stream reaches, and we detected only trypanosomes, but never leeches, in one reach. Among the 70% (588 of 841) of capture occasions when we detected at least one parasite, trypanosome-only infections were most common (54%), followed by coinfection with leeches and trypanosomes (39%) and leech-only infections (6%).

#### Parasite distribution at the landscape scale

**Figure 2:**
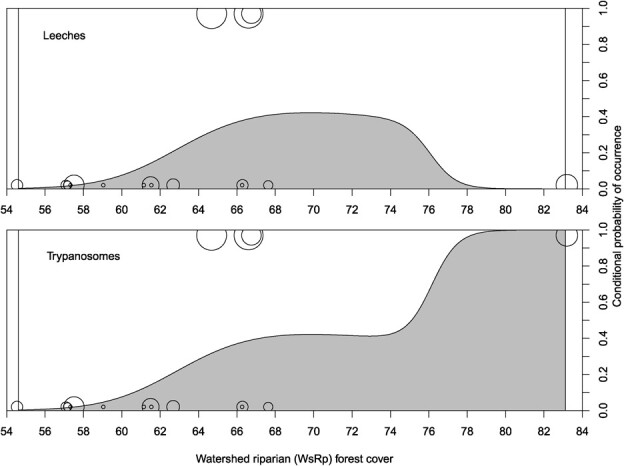
Conditional probability of parasite occurrence within a stream reach as a function of riparian forest within the contributing upstream watershed (WsRp), based on a sample of 17 stream reaches occupied by hellbenders (*Cryptobranchus alleganiensis*) in Virginia, USA, 2013–2016. Note that estimates should be interpreted with caution as conditional density estimates are less reliable for values of forest cover that are poorly represented in the sample. Open circles represent individual stream reaches, their relative size represents the log-transformed sample size (*ln* (*n* + 1)) of hellbender captures used to survey for parasites and the position of circles on the y-axis indicates whether each parasite was detected (1) or not (0) in the reach. Note that the difference in conditional probability of occurrence for leeches and trypanosomes is driven by a single stream reach (83% WsRp forest) where trypanosomes were detected but leeches were not.

In general, the probability of each parasite occurring in a stream reach increased as WsRp forest increased. While leeches were suggested to occupy a slightly narrower gradient of WsRp forest cover (65–75%) than trypanosomes (> 64%; Fig. 2), the difference in estimated distribution of each parasite was driven entirely by the status of one stream reach (R17) representing the highest levels of WsRp forest in our sample and where we detected trypanosomes but not leeches (Fig. 2). Because a) conditional density estimates are known to be less reliable for levels of the independent variable that are poorly represented in a sample (e.g. WsRp forest > 70%; Fig. 2, b) unpublished *ex situ* experimental data collected by one of us (W.A. Hopkins) indicates that leeches can function as a vector of trypanosome transmission from one hellbender to another and c) leeches have seasonal attachment dynamics to their hellbender hosts and can thus evade detection based on survey timing (see below), we interpret the differences in plots with considerable skepticism and suspect both parasites occupied a largely identical gradient of WsRp forest in our system.

#### Leech prevalence within reaches

**Figure 3:**
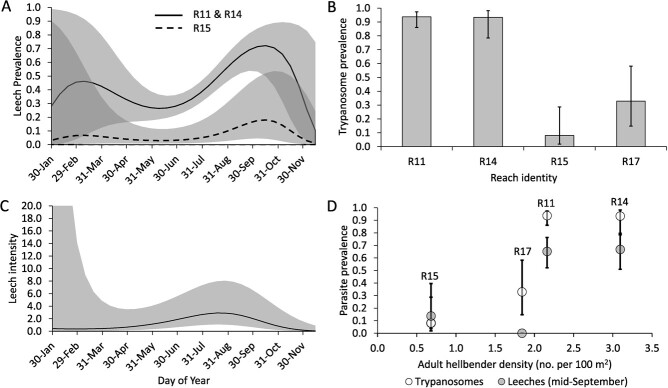
Patterns of leech (A,C,D) and trypanosome infection (B,D) in hellbenders (*Cryptobranchus alleganiensis alleganiensis*) from four stream reaches (R11, R14, R15 and R17) in Virginia, USA, 2013–2016. Note that that seasonal peaks in leech prevalence (A) lag slightly behind peak leech intensity (B) and that prevalence of both parasites was positively correlated with host density in 2015 (estimated by Bodinof Jachowski and Hopkins, 2018) within a reach. Solid lines, bar heights and points represent mean estimates based on top-ranking models and shaded areas and error bars represent 95% confidence intervals.

After subsetting our data to include only captures from the three reaches (R11, R14, R15) where leeches were detected we were left with 651 capture occasions for modeling leech prevalence (Table 1). Our top-ranked model (~Season + Reach; Table 2) provided strong evidence that prevalence of leech infections varied predictably with season and among the three reaches where leeches were confirmed to occur. The best supported form of DOY (fourth-order polynomial; *w_1_* > 0.99*)* suggested a possible peak in leech prevalence during early spring (Feb) and indicated a clear peak in early fall (Sep; Fig. 3A). Among reaches, prevalence of leech infections was nearly identical in reaches 11 and 14, where it was five- to nine-times higher than in reach 15. *Post-hoc* examinations of our leech prevalence estimates alongside estimates of hellbender density from the same reaches (estimated in 2015 by Bodinof Jachowski and Hopkins, 2018) provided compelling evidence that leech prevalence generally increased as adult hellbender density within a reach increased (Fig. 3A,D).

**Table 2:** Ranking of candidate models used to investigate factors associated with the parasitic infection (leeches and trypanosomes) in hellbenders (*Cryptobranchus alleganiensis*) from Virginia, USA, 2013–2016

Model	K	LL	AICc	Δ AICc	*w_i_*	
*Leech Prevalence*					
Season^a^ + Reach + ID	8	−397.16	810.56	0.00	1.00	
Season^a^ + ID	6	−409.62	831.38	20.82	0.00	
Season^a^ + WsRp Forest + ID	7	−409.00	832.19	21.63	0.00	
					
*Leech Intensity*					
Season^b^ + WsRp Forest + ID	7	−665.34	1345.11	0.00	0.47	
Season^b^ + ID	6	−666.70	1345.73	0.63	0.34	
Season^b^ + Reach + ID	8	−665.17	1346.90	1.80	0.19	
					
*Trypanosome Prevalence*					
Reach + ID	5	−301.75	613.57	0.00	1.00	
WsRp Forest + ID	3	−335.22	676.47	62.90	0.00	
ID	2	−347.36	698.74	85.16	0.00	

For each analysis models are arranged from best to least supported according to Akaike Information Criterion (AICc). K = number of estimated parameters; LL = Log-likelihood; *w_i_* = relative weight of evidence; WsRp = watershed riparian area; Reach = factor representing sampling reach, ID = random effect for individual.

a ^a^Season was modeled as a fourth-order polynomial form of day of year

b ^b^Season was modeled as a third-order polynomial form of day of year

#### Leech intensity within reaches

After subsetting our data to include only non-zero values of leech abundance, we were left with 271 capture occasions for modeling variation in leech infection intensity. Our analysis provided strong evidence that the intensity of leech infections varied seasonally. The best supported form of DOY (third-order polynomial; *w_1_ =* 0.77*)* indicated that the intensity of leech infections gradually increased throughout spring, peaked mid-August (mean intensity = 2.9 leeches [1.1–8.0]) and declined as winter approached (Fig. 3C). While our top-ranked model included a term for WsRp forest (Table 1), confidence intervals associated with its effect size overlapped zero (WsRp forest: β = 0.22 [−0.04–0.48]). Given the considerable uncertainty about the effect of WsRp forest on leech intensity, we do not discuss it further.

#### Trypanosome prevalence within reaches

Focusing on capture records from the four reaches (R11, R14, R15, R17) where trypanosomes were detected yielded 717 capture occasions for modeling trypanosome prevalence. Unlike leech infections, we found no evidence of seasonal variation in trypanosome prevalence (Intercept-only model; *w_1_* = 0.59). However, similar to leech infections, we detected strong evidence that the prevalence of trypanosome infections varied among the four reaches where the parasite was detected (Table 2); ranging from just 8% of occasions in R15 to > 90% of occasions in R11 and R14 (Fig. 3B). A *post-hoc* examination of trypanosome prevalence estimates for each reach alongside estimates of hellbender density from the same reaches (estimated in 2015 by Bodinof Jachowski and Hopkins, 2018) provided compelling evidence that prevalence of trypanosome infections generally increased as adult hellbender density in a reach increased (Fig. 3B,D).

### Effects of Land Use and Parasites on Physiological Condition

#### Body condition

**Figure 4:**
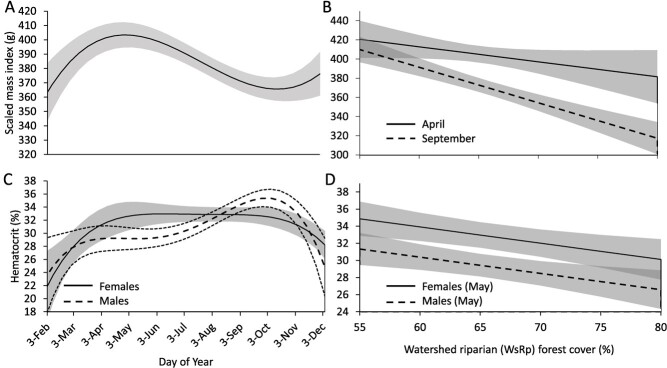
Estimated effects of season (A,C), riparian forest cover within the contributing watershed (B, D) and sex on physiological condition indices of hellbenders (*Cryptobranchus alleganiensis*) from Virginia, USA, 2013–2016. Scaled mass index (A-B) is a measure of body condition, defined here as estimated mass of an individual assuming it were 40 cm total length. Hematocrit (C-D) refers to the proportion of blood composed of red blood cells. Seasonal variation in scaled mass (A) and hematocrit (C) are visualized assuming average (65%) levels of WsRp riparian forest cover. Solid and long-dashed lines represent mean estimates and shaded areas and short-dashed lines represent 95% confidence intervals based on top-ranked models.

After discarding records from adults of unknown sex we were left with 814 samples from 383 individuals (187 females; 196 males) for modeling SMI. Model ranking provided strong evidence that hellbender body condition was better predicted by WsRp forest cover than current state of parasitic infection, and more specifically, that WsRp forest or some correlate, modulated the nature of seasonal fluctuations in body condition (Table 3). The best supported form of DOY (third order polynomial; *w_1_ =* 0.61) indicated that, under average conditions in our system (65% WsRp forest), body condition reached an annual high in late April, declined to a low point in mid-September and gradually increased throughout winter and spring (Fig. 4A). While the general timing of seasonal highs and lows in body condition was consistent across our land cover gradient, the absolute difference between high and low points increased as WsRp forest increased (Fig. 4B). For example, hellbenders subject to relatively low (55%) WsRp forest cover experienced negligible (2%; ~ 9 g) decline in body condition between April and September while hellbenders subject to moderate (65%) to high (80%) levels of WsRp forest exhibited an 8% (~ 31 g) to 17% (~64 g) decline in body condition, respectively (Fig. 4B). Additionally, in any given season, body condition was negatively correlated with WsRp forest (Fig. 3B), most notably in September, when scaled mass in areas subject to just 55% WsRp forest was approximately 11% (37 g) higher than that of hellbenders subject to 65% WsRp forest and 30% (96 g) higher than that of hellbenders subject to 80% WsRp (Fig. 4B). Though our top-ranking model included a term for sex, confidence intervals associated with its effect size broadly overlapped zero (β_male_ = 2.95 [−6.44–12.36]), indicating considerable uncertainty about sex effects on hellbender body condition. As such we ignored sex effects and relied solely on other terms in the model for inference.

**Table 3:** Ranking of candidate models used to investigate factors associated with body condition, hematocrit and white blood cell (WBC) profiles of hellbenders (*Cryptobranchus alleganiensis*) from Virginia, USA, 2013–2016

Model	K	LL	AICc	Δ AICc	*w_i_*
*Body Condition*					
(Season^b^ x WsRp Forest) + Sex + ID	11	−4187.29	8396.91	0.00	0.95
(Season^b^ x Sex) + WsRp Forest + ID	11	−4190.23	8402.80	5.89	0.05
Season^b^ + ID	6	−4226.28	8464.66	67.76	0.00
(Season^b^ x Sex) + ID	10	−4225.38	8471.03	74.13	0.00
(Season^b^ x Sex) + Infection + ID	13	−4222.88	8472.21	75.30	0.00
(Season^b^ x Infection) + Sex + ID	19	−4219.40	8477.75	80.84	0.00
					
*Hematocrit*					
(Season^a^ x Sex) + WsRp Forest + ID	13	−1531.13	3089.03	0.00	0.84
(Season^a^ x Sex) + Infection + ID	14	−1531.97	3092.83	3.79	0.13
(Season^a^ x Sex) + ID	12	−1535.34	3095.33	6.30	0.04
Season^a^ + ID	7	−1545.36	3104.96	15.93	0.00
(Season^a^ x WsRp Forest) + Sex + ID	13	−1540.28	3107.34	18.31	0.00
(Season^a^ x Infection) + Sex + ID	15	−1545.06	3121.13	32.10	0.00
*WBC PC 1 (+N:L ratios, + Eosinophils)*					
(Season^b^ x Sex) + Infection + ID	14	−543.94	1117.27	0.00	0.59
Season^b^ + ID	7	−552.28	1118.93	1.66	0.26
(Season^b^ x WsRp Forest) + Sex + ID	13	−546.99	1121.18	3.91	0.08
(Season^b^ x Sex) + ID	13	−547.94	1123.08	5.81	0.03
(Season^b^ x Sex) + WsRp Forest + ID	12	−549.35	1123.72	6.46	0.02
(Season^b^ x Infection) + Sex + ID	15	−546.67	1124.93	7.66	0.01

White blood cell parameters were reduced to a single independent response variable (PC 1) using principal components analysis prior to analysis, which primarily captured variance drive by co-elevated neutrophil:lymphocyte (N:L) ratios and eosinophil levels. Models are arranged from best to least supported according to Akaike Information Criterion (AICc). K = number of estimated parameters; LL = Log-likelihood; *w_i_* = relative weight of evidence; WsRp = watershed riparian area; ID = random effect for individual.

a ^a^ Season was modeled as a third-order polynomial

b ^b^Season was modeled as a fourth-order polynomial

#### Hematocrit

Discarding records from adults of unknown sex yielded 488 samples from 255 individuals (120 females; 135 males) for modeling variation in hematocrit. Our results provided strong evidence that hematocrit was most influenced by season and sex and was more closely associated with WsRp forest than current state of infection. Males and females exhibited distinctly different hematocrit profiles over an annual timescale (Table 1). The best supported form of DOY (fourth order polynomial; *w_1_* = 0.98) indicated that, in both sexes, hematocrit reached an annual low during winter (Dec-Feb) and increased gradually throughout spring. While hematocrit of females peaked in early summer (May–June) and began to decline in late August, hematocrit of males continued to increase throughout summer and did not begin to decline until late October (Fig. 4C). In May specifically, mean hematocrit of females (33% [31–34]) was about three percentage points higher than mean levels of males (29% [28–31]) while in early September mean hematocrit of males (35% [34–36]) was about two percentage points higher than mean levels of females (33% [32–34]). Additionally, we observed strong evidence that hematocrit was negatively correlated with WsRp forest, though the effect size was relatively small. For example, hematocrit declined by an absolute value of 0.95 percentage points for every 5% increase in WsRp forest. As such, during any given season, the hematocrit of hellbenders subject to just 55% WsRp forest was estimated to be 5 percentage points higher than that of hellbenders subject to 80% WsRp forest, once sex effects were accounted for (Fig. 4D).

#### WBC parameters

Discarding records from adults of unknown sex yielded 319 samples from 209 individuals (105 females; 104 males) for modeling WBC profiles. Among these samples lymphocytes were the most common type of WBC observed (median = 69%, range = 30–97%), followed by neutrophils (median = 22%, range = 1–60%), eosinophils (median = 6%, range = 0–24%), basophils (median = 1%, range = 0–8%) and monocytes (median = 0%, range = 0–2%; Supplementary Table S1). Due to the rarity of basophils and monocytes, we did not consider them in our analyses. Mean observed N:L ratios were 0.44 ± 0.02 SE, however, the range of N:L ratios observed during our study spanned an order of magnitude (range = 0.01–1.97). Neutrophil-to-lymphocyte ratios were strongly and equally correlated with % neutrophils (r = 0.95) and % lymphocytes (r = −0.93), confirming that variation in N:L ratios was primarily driven by concurrent changes in representation of both cell types. Eosinophils were moderately correlated with lymphocytes (r = −0.63) and slightly correlated with neutrophils (r = 0.40) and N:L ratios (r = 0.44).

**Figure 5:**
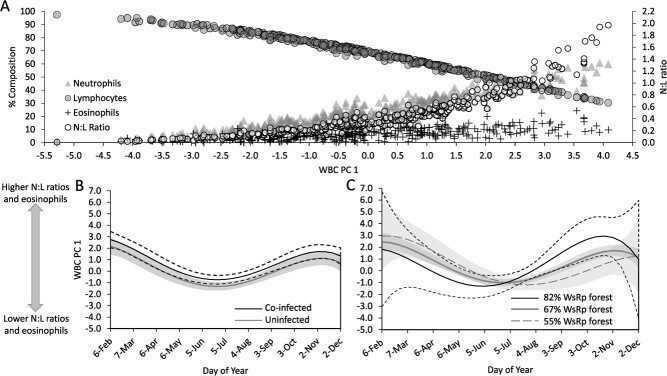
Associations between white blood cell principal component one (WBC PC 1) and A) four interdependent white blood cell parameters collected from hellbenders (*Cryptobranchus alleganiensis*) in Virginia, USA, 2014–2015. Note that PC 1 primarily explains variation in neutrophil-to-lymphocyte (N: L) ratios which were often, but not always, correlated with eosinophil levels. Lower panels illustrate estimated effects of (B) season and parasitic infection based on a top-ranking model or (C) season and riparian forest cover within the contributing watershed (WsRp forest) according to a third-ranked model. Note that both models indicate that N:L ratios peaked late-fall through winter and were minimal during summer but differ regarding timing and intensity of seasonal variation in PC 1. Solid and long-dashed lines represent mean estimates and shading and short-dashed lines represent 95% confidence intervals.

The first principal component in our WBC PCA explained the majority (82%) of variation in WBC parameters and thus was the only principal component retained for further analysis. Variable loading scores indicated that the PC1 exhibited a moderately strong positive correlation with N:L ratios (loading = 0.54) and % neutrophils (loading = 0.53), a moderate positive correlation with % eosinophils (loading = 0.36) and a moderately strong negative correlation with % lymphocytes (loading = −0.54). Thus, we interpreted PC 1 to largely represent variation due to co-elevated N:L ratios and eosinophil levels, where large values of PC 1 were associated with individuals that exhibited both elevated N:L ratios and elevated eosinophil levels and small values were associated with the opposite scenario (Fig. 5A). For reference, PC 1 values ranged from −5.2 to 4.0, where values ≤ −1.0 were always associated with N:L ratios ≤ 0.25 and values ≥2.5 were always associated N:L ratios ≥0.85 (Fig. 5A).

The weight of evidence in our main analysis (Table 1) indicated that WBC PC1 varied predictably in response to season and parasitic infection. The best supported form of DOY (fourth order polynomial; *w_1_* = 1.00) suggested WBC PC 1 reached an annual low in mid-summer and annual high from late fall through winter (Fig. 5B). Given the correlation between N:L ratios and PC 1, N:L ratios were about 4-fold higher, and eosinophil levels were about 2-fold higher, in mid-winter (mean N:L ratios ~ 0.8; mean eosinophils ~ 10%) relative to mid-summer (mean N:L ratio ~ 0.2; mean eosinophils ~ 5%; Fig. 5). Hellbenders infected with at least one parasite had slightly higher PC 1 scores than uninfected individuals, but we observed no evidence that WBC profiles differed between single parasite infections (β_single_ = 0.59 [0.19–0.9]) and co-infections (β_co-infected_ = 0.68 [0.25–1.11]). Considering the correlation we observed between PC 1 and each WBC parameter (Fig. 5A), our findings indicated that infected individuals exhibited 30% higher N:L ratios and a single percentage point increase in eosinophil levels relative to uninfected individuals. The interaction between sex and season in our top-ranked model was not well supported (β_male_ = −0.23 [−0.65–0.20]; β_male*DOY_ = −0.56 [−1.21–0.08]; β_male*DOY2_ = 0.29 [−0.37–0.97]; β_male*DOY3_ = 0.12 [−0.06–0.31]; β_male*DOY4_ = -0.06 [−0.21–0.10]) and thus is not discussed further.

Though considerably less supported, two additional models fell within our confidence set of models describing variation in WBC PC1 (Table 1). Notably, the seasonal fluctuations in WBC PC1 indicated by the second and third ranking models were largely similar to those described under our top-ranked model. However, our second-ranked model (~Season) suggested WBC PC1 was otherwise similar among stream reaches. In contrast, our third-rank model (~Season*WsRp Forest + Sex) provided moderate evidence that forest cover may have mediated seasonal fluctuations in WBC profiles, such that WBC parameters were similar among stream reaches early in the year (Feb-July), but PC 1 scores increased as WsRp forest increased, particularly between late July and late November (Fig. 5C). Given the correlation between PC 1 and WBC parameters (Fig. 4A), the effect size of WsRp forest on PC 1 corresponded to an approximate 1.5-fold increase in N:L ratios and 1 percentage point increase in eosinophils for every 10% increase in forest cover throughout the watershed riparian area. Sex effects in our third-rank model were not well supported (β_male_ = −0.11 [−0.50–0.28]) and thus not discussed further.

## Discussion

Riparian forest cover was predictive of both occurrence and prevalence of leech and trypanosome infections in hellbenders from a given stream reach. Relationships between land use and parasite distributions in amphibians are highly variable, given the diversity of host specificity and life cycles among parasites (Koprivnikar *et al.*, 2012). In anuran systems abundance of trematode and nematode parasites may increase (McKenzie, 2007) or decrease (Hartson *et al.*, 2011) following loss of forest cover, with differences generally attributed to how intermediate and/or determinant host abundance is affected by land use change (Hudson *et al.*, 2006). The positive association between riparian forest cover and parasite status described herein is remarkably consistent with the positive associations between forest cover and metrics of hellbender status that have been previously described (Bodinof Jachowski and Hopkins, 2018; Bodinof Jachowski *et al.*, 2016; Hopkins *et al.*, 2023) and suggest that the distribution of our focal parasites was largely determined by the underlying patterns of local abundance of hellbender hosts which respond positively to riparian forest cover (Bodinof Jachowski and Hopkins, 2018). Our findings are consistent with patterns of parasitism in some other salamanders (*Plethodon cinereus,*Davis *et al.* 2016) and, when viewed collectively, are indicative of classic density-dependent parasite transmission (Dietz, 1988). We are thus led to conclude that, in our system, stream reaches of highest abiotic quality not only harbor the highest density of hellbenders, but also present the greatest risk of hellbenders contracting leech and trypanosome infections. Though more work is needed to investigate the potential of our focal leech and trypanosome to use alternative vertebrate hosts, our findings provide evidence that hellbender density may be a limiting factor to their geographic distributions (Arneberg *et al.*, 1998). If some minimum level of local hellbender density is necessary to support each parasite at the scale of a stream reach, both parasites may have historically exhibited broader geographic distributions than we detected but are now restricted to areas where hellbender density has remained sufficiently high to facilitate their persistence.

In reaches where parasites occurred, leech prevalence varied seasonally while trypanosome prevalence did not. Parasitic infections often vary seasonally, though the apparent drivers or timing of peak prevalence or intensity is highly variable across parasite taxa (Poulin, 2007). We suspect that patterns of leech prevalence in our system were primarily driven by reproductive phenology of leeches and their hellbender hosts. For example, *P. appalachiensis* exchange spermatophores and externalize eggs in May, after which young hatch but remain attached to the parent who provides care until detachment in August (Hopkins *et al.*, unpublished data*)*. Thus, peak leech intensity during late August (Fig. 3C) and peak leech prevalence that followed shortly thereafter (Fig. 3A) were likely the result of detachment and dispersal of offspring, which is consistent with anecdotal observations that the highest intensity leech infections were characterized predominantly by young-of-the-year leeches (pers. obs). Peak leech infection intensity also coincided with the brief period when hellbenders interact with each other for spawning, suggesting that increases in rates of host movement and contact with other hosts or rock surfaces harboring leeches may have contributed to patterns of leech infection. Notably, we regularly observe leeches attached to the underside of rocks used for cover by hellbenders, suggesting that seasonal lows in leech prevalence (Fig. 3A,C) are likely associated with periods of detachment from hosts and attachment to stream substrates. In contrast to leech infections, trypanosome prevalence was consistently high year-round. Because the lifecycle of this trypanosome is unknown, we were unable to determine if the lack of seasonality in trypanosome prevalence was the result of long infection duration or frequent re-infection. In mammalian systems the duration of trypanosome infections may last months to years in some hosts while disease progresses through distinct stages with associated changes in parasite infection intensity (Kipkorir *et al.*, 2021; MacLean *et al.*, 2010; Odiit *et al.*, 1997). Thus, future efforts to understand the basic biology of this trypanosome would enhance our ability to understand the progression of trypanosome infections in hellbenders and lend insight into the potential long-term implications of chronic infection.

Living in heavily forested stream reaches with an elevated risk of parasitism by leeches and trypanosomes was associated with relatively high N:L ratios and eosinophil levels, lower mean hematocrit levels and lower mean body condition in hellbenders. In contrast, hellbenders exposed to the highest levels of anthropogenic land use appeared to experience a potential benefit in the form of ‘release’ from leeches and trypanosomes and a possibly related increased ability to invest in energy stores (e.g. improved physical body condition). While these patterns align with our predictions under the hypothesis that hellbender condition would respond to parasitism, the proximate mechanism responsible for the variation in each condition index along our gradient of WsRp forest remains unclear because riparian forest cover, water quality, host density and parasite prevalence were all positively correlated in our system, as discussed in more detail below.

White blood cell profiles of hellbenders were most likely a cumulative response to predictable seasonal fluctuations in stream temperature and spatiotemporal patterns of parasitic infection. Most of the variation in hellbender WBC profiles was explained by changes in the proportional representation of just three cell types (neutrophils, lymphocytes and eosinophils). Lack of a complete WBC count precluded our ability to distinguish between elevated N:L ratios that were the result of increased neutrophil production (e.g. in response to infection or physiological stress; Davis *et al.*, 2008) and those that were simply an artifact of downregulation in lymphocyte production (e.g. in response to cooler temperatures; Maniero and Carey, 1997). However, considering the context of our study provides some indication that both mechanisms were at play. Because neutrophils are one of the first phagocytic cells to respond to stress or infection and eosinophils play an important role in the inflammatory response and defense against parasites specifically (Allender and Fry, 2008), concurrent increases in N:L ratios and eosinophils is consistent with an immune response to parasitism in hellbenders (Hopkins *et al.*, 2016). Thus, the commonality of co-elevated N:L ratios and eosinophils that we observed suggests that parasitism, as opposed to chronic physiological stress, was an important driver of variation in N:L ratios during any given season. Additionally, the biologically relevant (i.e. 4-fold) increase in hellbender N:L ratios following the transition from summer to winter in our system is consistent with our hypothesis that lymphocyte production would curtail following exposure to cooler temperatures, as occurs in other amphibians (Maniero and Carey, 1997). However, because cooling temperatures were somewhat confounded with increasing parasitic infections (Fig. 3), the degree to which parasites may have enhanced otherwise typical seasonal variation in hellbender N:L ratios is unclear. We interpret the model selection uncertainty in our analysis of WBC PC 1 as indication that our data were consistent with two equally-likely scenarios: one where most of the annual variation in N:L ratios was thermally driven and parasitic infection was associated with a relatively small effect size that was consistent year-round (Fig. 5 A,B) or one where thermally-driven variation in N:L ratios was less severe but the immune response to parasitism was seasonally-constrained between fall and winter and increased in intensity as WsRp forest (a proxy of sub-population density and parasite prevalence) increased (Fig. 5C). While both scenarios are consistent with our hypothesis that hellbenders physiologically respond to parasitic infection, the intensity of the physiological response and the temporal window each year in which it occurs differs. Future efforts to characterize hellbender WBC profiles in systems lacking leeches and trypanosomes could help clarify the degree to which annual variation in hellbender WBC profiles described here were thermally-driven as opposed to a response to temporal parasite dynamics.

In contrast to our predictions, hellbender WBC profiles provided no evidence that relatively poor habitat quality resulted in chronic physiological stress. Because N:L ratios correlate with circulating levels of glucocorticoid hormones they are increasingly relied on as a measure of physiological stress in vertebrates, including amphibians (Davis *et al.*, 2008). While we did not evaluate correlations between N:L ratios and water quality directly, WsRp forest is positively correlated with water quality in our system (Supplementary Figure S1). Thus, the lack of a negative association between WsRp forest and WBC PC1 in our study contrasts somewhat with findings of Litmer *et al.* (2020), who reported an increase in hellbender N:L ratios in response to degraded water quality. One possible explanation for this contrast could be that parasite effects simply overwhelmed any effects of altered abiotic habitat conditions in our systems. For example, we found that N:L ratios and eosinophils were often co-elevated, which is indicative of parasitic infection (Hopkins *et al.*, 2016), while a typical response to glucocorticoid secretion is characterized by increasing N:L ratios but a decline in eosinophils (Davis *et al.*, 2008; Falso *et al.*, 2015). Otherwise, mean monthly N:L ratios observed in our study between April and August (range: 0.28–0.66; Supplementary Table S1) were similar to those reported for most sites sampled by Litmer *et al.* (2020), which only rarely exceeded 1.0, and thus collectively suggest that few if any of the sub-populations sampled in either study exhibited signals of true physiological stress (i.e. N:L ratio > 1.0; Davis and Maerz, 2022).

During any given season, increasing WsRp forest was associated with a slight decline in hematocrit. In general, hematocrit is positively correlated with the oxygen carrying capacity of blood and seasonal fluctuation in hematocrit is generally expected to facilitate shifting metabolic demands in amphibians (Harris, 1972). However, during any given season, elevation in hematocrit can signify increased physiological stress like that associated with handling or dehydration (Barsotti *et al.*, 2019), and reduction in hematocrit can accompany infestations with blood associated parasites (Toque *et al.,* 1993). Though effect sizes were relatively small (Fig. 4D), our findings are consistent with predictions under our hypotheses that hellbender hematocrit would respond negatively to parasitic infection as well as habitat degradation. However, the confoundance between abiotic habitat quality and parasite occurrence in our system ultimately precluded our ability to determine which stressor was the dominant driver of spatial variation in hematocrit or whether both were important.

Though our primary interest was to understand how hematocrit responded to proposed stressors, a novel finding from our study was evidence of sex-specific differences in annual hellbender hematocrit profiles. The difference in hematocrit profiles for males and females that we detected (Fig. 4C) may be due to the disassociation between timing of peak metabolic investment in annual reproduction for each sex (Acuña, 1974). Both males and females exhibited lowest hematocrit in winter when and activity is generally minimal (Bodinof *et al.*, 2012*b*), and dissolved oxygen is at its highest due to low water temperature. Male hematocrit peaked between September and October, which aligns with spawning, brooding eggs and frequent aggressive interactions with conspecifics (Unger *et al.*, 2020). In contrast, female hematocrit peaked in April and remained stable over summer, which aligns with the period when females are presumably focused on acquiring energy to invest in oocyte development. In some lizards, red blood cell disintegration can accompany oocyte development in females and exhaustion following breeding investment in males (Banerjee and Banerjee, 1969), which could explain why female hematocrit began declining in late August (when gravidity is most evident; pers. obs.) while male levels began declining in November (approximately when hatching occurs; Fig. 4C). Notably, previous work in our system failed to detect sex differences in hematocrit, likely because animals in that study were sampled during a brief period in early August, when our data suggest sex differences do not exist (Fig. 4C). Otherwise, hematocrit levels reported by Hopkins *et al.* (2016) were consistent with values reported herein.

During any given season, hellbender body condition declined in response to increasing WsRp forest, hellbender sub-population density and associated increase in the risk of parasitic infection. These findings are consistent with those from many fish systems, where body mass is negatively correlated with population density (i.e. self-thinning), though mechanisms often remain unclear (Dunham and Vinyard, 1997). Poorer body condition of hellbenders in more heavily forested stream reaches could indicate direct physiological effects of frequent parasitic infection or, alternatively, be a response to higher rates of host movement and host-to-host contact that are indirect responses to limited resource availability and host crowding (see Sánchez *et al.*, 2018). Additionally, nutrient enrichment and altered thermal regimes that often accompany loss of forest cover have been associated with an increased prey base and growth rates of aquatic salamanders (Barrett *et al.*, 2010; Bumpers *et al.*, 2015; Guzy *et al.*, 2021), suggesting that higher body condition in more degraded stream reaches could be attributable to altered energy flow and higher biomass of hellbender prey in those stream reaches (Bumpers *et al.*, 2017).

The loss of seasonal variation in body condition that accompanied declining forest cover highlights potentially important consequences of anthropogenic land use for hellbenders. Among amphibians and fish, decreases in body condition during the spawning season are expected due to oviposition in females, increased metabolism and decreased feeding for both sexes (Ryser, 1989; Brown and Murphy, 2004). Concordantly, temporal variation in body condition of hellbenders subject to relatively high forest cover (> 65% WsRp) largely reflected reproductive phenology. For example, hellbenders exhibited peak body condition in early summer, presumably when foraging conditions were optimal and poorest body condition in late September, just after spawning. In contrast, hellbenders subject to < 65% WsRp forest exhibited static body condition year-round, despite exhibiting relatively high body condition on average. The virtual absence of seasonal body condition fluctuations in our most degraded stream reaches was especially surprising given that amphibians in better body condition generally invest more resources in reproduction (Castellano *et al.*, 2004). Our findings suggest that hellbender occupying more degraded stream reaches were either better equipped to rapidly compensate for declines in body condition following spawning and/or they invested less in spawning or experienced lower spawning success altogether. Hopkins *et al.* (2023) reported a marked increase in probability of hellbender nest failure, specifically due to whole-clutch filial cannibalism, along a gradient of declining WsRp forest in our very study system. Because higher rates of egg cannibalization shortly after oviposition would essentially offset any body-condition-based signature of spawning investment in the sub-population, increasing rates of egg cannibalism offers a particularly compelling explanation for the reduced degree of annual fluctuation in body condition that we describe along a declining forest cover gradient. As such, we consider our findings to be largely corroborative of growing evidence that reductions in riparian forest cover can act as an overarching driver of hellbender declines, specifically through the mechanisms of reduced reproductive success (Bodinof Jachowski and Hopkins, 2018; Hopkins *et al.*, 2023).

## Conservation Implications

Habitat loss and disease are two of the greatest threats facing amphibians and predicting how they interact in nature is a growing challenge for conservation (Bower *et al.*, 2019). Viewed collectively, our findings provide multiple lines of evidence that hellbenders respond physiologically to both leech and/or trypanosome infections and anthropogenic land use, but that these stressors may rarely co-occur in our system for extended periods because land use alteration can lead to localized extirpation of parasites via declining host density. While our findings indicate that hellbenders respond physiologically to parasitic infection, or some correlate, the relatively robust population density and demographic characteristics of the most heavily parasitized sub-populations that we examined (see Bodinof Jachowski and Hopkins, 2018; Hopkins *et al.*, 2023) suggests that any costs associated with this response may be negligible when abiotic habitat quality is high. However, we urge caution against assuming that our focal parasites are entirely benign, given that we studied their effects on hosts during a relatively narrow temporal window relative to hellbender longevity (25+ years) and because future environmental change might limit the ability of hellbenders to tolerate some parasites. As such, we consider the relatively high rate of leech prevalence between September and November to be of special interest given that it coincides with hellbender spawning and egg brooding, and parental care is critical for nest success (Hopkins *et al.*, 2023). A limitation of our study was the absence of parasites from our most degraded stream reaches, which precluded our ability understand how habitat degradation and parasites might interactively affect hellbenders.

Though our findings suggest that individual hellbenders exposed to lower levels of WsRp forest, and thus reduced habitat quality, may experience an improved ability to allocate resources to energy storage or possibly somatic growth (see maximum body sizes reported by Bodinof Jachowski and Hopkins, 2018), our prior work on hellbender population characteristics and reproduction (Bodinof Jachowski and Hopkins, 2018, Hopkins *et al.*, 2023) suggests that these individual-level advantages are unlikely to scale up to benefit the population. Because hellbender clutch size is known to increase with female body size (Topping and Ingersol, 1981), improved body condition or larger overall body sizes might be associated with higher fecundity in degraded habitats. While improved fecundity would normally be beneficial, land use alterations in our system have yielded chronically degraded stream conditions, low adult densities and exceptionally high rates of whole-clutch filial cannibalism that appear to be a limiting factor for population growth, regardless of mean clutch size (see Hopkins *et al.*, 2023). Given the current context, we speculate that large adults from degraded stream reaches in our system, may carry great potential to contribute to population and species recovery as either a source of eggs for *ex situ* head-starting programs or as breeding stock for captive propagation and translocation programs, which are a rapidly growing tools for hellbender recovery (Ettling *et al.*, 2017; Settle *et al.*, 2018).

## Supplementary Material

Web_Material_coad101
