# Supplementary material for: The habitat quality paradox: loss of riparian forest cover leads to decreased risk of parasitism and improved body condition in an imperiled amphibian
Source: Conserv Physiol. 2024 Jan 28;12(1):coad101. doi: 10.1093/conphys/coad101 (PMC10823334; doi:10.1093/conphys/coad101)
Supplement: Web_Material_coad101 [file web_material_coad101.pdf]

Supplemental Information A

Quantifying hematocrit

Immediately after blood collection, we partially filled two 75 µL heparinized hematocrit tubes with whole blood for measuring hematocrit and trypanosome screening. Hematocrit tubes were stored on ice, transported to the laboratory and centrifuged at 5*g* for 5 min within 10 h of collection. We quantified hematocrit (%) for each hellbender using a standard hematocrit capillary tube reader (McCormick Scientific, St. Louis, MO, USA). We used the average value of hematocrit based on replicate tubes collected during each capture event in our analyses.

We prepared blood smears to assess WBC profiles only for individuals captured during a 10-month period of our study (20 Oct 2014 - 21 Aug 2015). For these samples we made duplicate smears from whole blood at the same time that hematocrit tubes were being filled in the field, using a standard two-slide technique. We air dried slides and stored them dry until fixing and staining with a Wright-Giemsa stain (Camco Quik Stain II) within three weeks of collection.

After measuring hematocrit in the lab, we prepared buffy-coat smears (Hopkins *et al.*, 2016) for trypanosome screening. Briefly, this involved using the visible layer of WBC (buffy coat) in the hematocrit tube after centrifugation and approximately 2-3 µL of plasma (to aid in evenness of smears) to create a smear using a standard two-slide technique. We air dried buffy coat smears and stored them dry until fixing and staining with a Wright-Giemsa stain (Camco Quik Stain II) within one to six weeks of collection.

Trypanosome screening

To determine whether hellbenders were infected with trypanosomes we examined buffy coat smears at x400 magnification. A pilot study indicated the probability of detecting at least one trypanosome after viewing 50 random fields of view from a buffy coat smear was 0.90 ± 0.02 SE, and thus the cumulative probability of detecting trypanosomes when present after

scanning two slides was ≥ 0.99. After confirming that detectability of trypanosomes was robust to observer differences, slides were examined by one of three trained individuals (C. Bodinof Jachowski, V. Alaasam, A. Blumenthal) that were blind to the identity of the slide being screened. Observers scanned 50 random fields of view per slide and only screened a second slide if trypanosomes were not detected in the first slide. We categorized trypanosome infections as a binomial outcome (infected or not-infected) since methods to quantify intensity of infections from buffy coat smears have not been validated; where we defined individuals as infected if at least one trypanosome was detected and uninfected if no trypanosomes were detected after scanning both buffy coat slides.

Quantifying white blood cell differentials

We examined whole blood smears at x400 magnification to determine differential WBC profiles of hellbenders. All slides were examined by a single observer (V. Alaasam) who was blind to the identity of each slide. At least 100 leukocytes were counted and only fields of view with even distributions of cells were used (Forzán *et al.,* 2017). Cells were identified as lymphocytes, neutrophils, eosinophils, basophils or monocytes following Turner (1988), Thrall *et al.,* (2004) and Campbell and Ellis (2007). We determined the proportion of each cell type by dividing the number of cells of that type by the total number of WBC counted. We calculated N:L ratios by dividing the proportion of WBCs categorized as neutrophils by the proportion of WBCs categorized as lymphocytes.

Quantifying body condition

We calculated body condition as a scaled mass index (SMI; Peig and Green, 2009). Briefly, scaled mass is interpreted as the estimated mass of an individual if it were of a reference structural size (Peig and Green, 2009; MacCracken and Stebbings, 2012). Because urodeles store considerable energy reserves in the tail we used total length to quantify structural size of

hellbenders (Fitzpatrick, 1976; Takahashi and Pauley, 2010) and calculated scaled mass as $\widehat{SM}_i = M_i \left[\frac{L_0}{L_i}\right]^b$ where M_i was mass (g) and L_i was total length (cm) of individual *i*, L_0 was our selected reference structural size (40 cm total length) and b was a scaling exponent. We calculated *b* by dividing the slope from a regression of $\log_{10}$(mass [g]) against $\log_{10}$(total length [cm]) by the Pearson's correlation coefficient (*r)* describing the relationship between log-transforms mass and total length (Peig and Green, 2009). We used data from all observations collected during our study (*n* = 841 to determine slope coefficients (2.99 ± 0.04 SE), *r* (0.95), and *b* (3.14).

Supplemental Table 1. Summary of annual variation in white blood cell (WBC) parameters of eastern hellbenders (*Cryptobranchus alleganiensis*) in southwest Virginia, USA. Values are reported as mean [range] of observed values.

Month	n	% Monocytes	% Basophils	% Eosinophils	% Lymphocytes	% Neutrophils	N:L Ratio
February	19	0 [0-0]	0 [0-0]	12 [6-23]	44 [20-61]	44 [30-73]	1.09 [0.28-1.97]
March	22	0 [0-2]	1 [0-3]	8 [0-18]	34 [10-56]	58 [34-85]	0.64 [0.12-1.36]
April	2	0 [0-0]	2 [2-2]	6 [6-6]	36 [35-38]	56 [54-57]	0.66 [0.62-0.69]
May	28	0 [0-1]	2 [0-6]	8 [2-24]	28 [13-56]	63 [32-79]	0.52 [0.17-1.68]
June	62	0 [0-1]	1 [0-8]	6 [0-23]	24 [6-52]	69 [39-90]	0.37 [0.06-1.33]
July	86	0 [0-1]	2 [0-8]	5 [0-18]	16 [2-37]	77 [49-94]	0.23 [0.02-0.74]
August	71	0 [0-1]	1 [0-7]	6 [0-20]	18 [1-47]	74 [44-97]	0.28 [0.01-1.07]
October	29	0 [0-1]	0 [0-2]	8 [2-16]	38 [15-57]	53 [35-82]	0.78 [0.19-1.58]
November	4	0 [0-0]	0 [0-0]	11 [6-16]	33 [27-38]	55 [47-62]	0.63 [0.44-0.80]
December	16	0 [0-0]	0 [0-1]	8 [3-14]	33 [18-58]	59 [34-73]	0.65 [0.25-1.70]

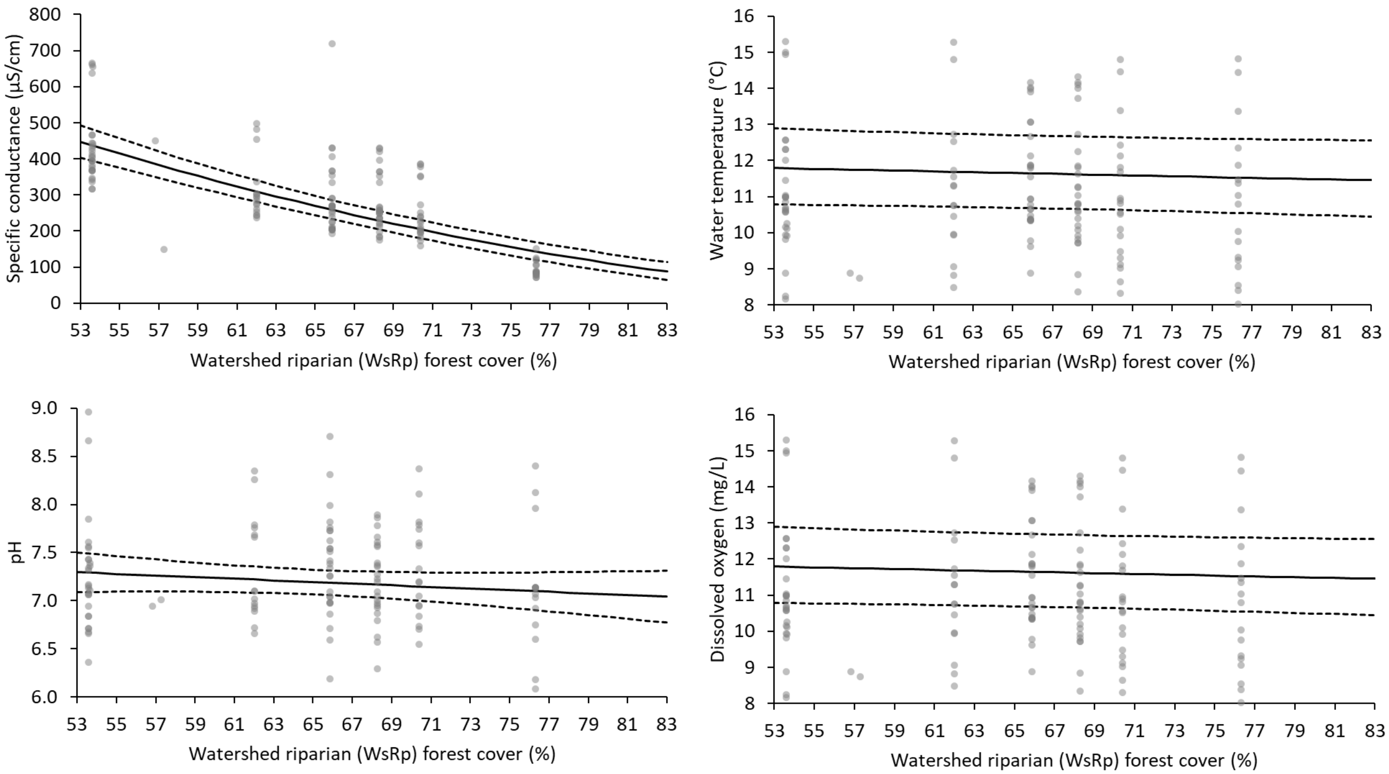

Supplemental Figure 1. Association between watershed riparian (WsRp) forest cover and four water quality metrics. Points represent observed data from 14 stream reaches in southwestern Virginia, USA, where hellbenders (*Cryptobranchus alleganiensis*) were surveyed between 2013 and 2016. Solid lines represent mean estimated effects from generalized linear mixed models where month was included as a random effect term to account for predictable seasonal variation in each response. Note that water temperature, dissolved oxygen, and pH varied little with land cover but that specific conductance (a measure of ionic concentration and impurities in stream water) decreased considerably as riparian forest cover increased.

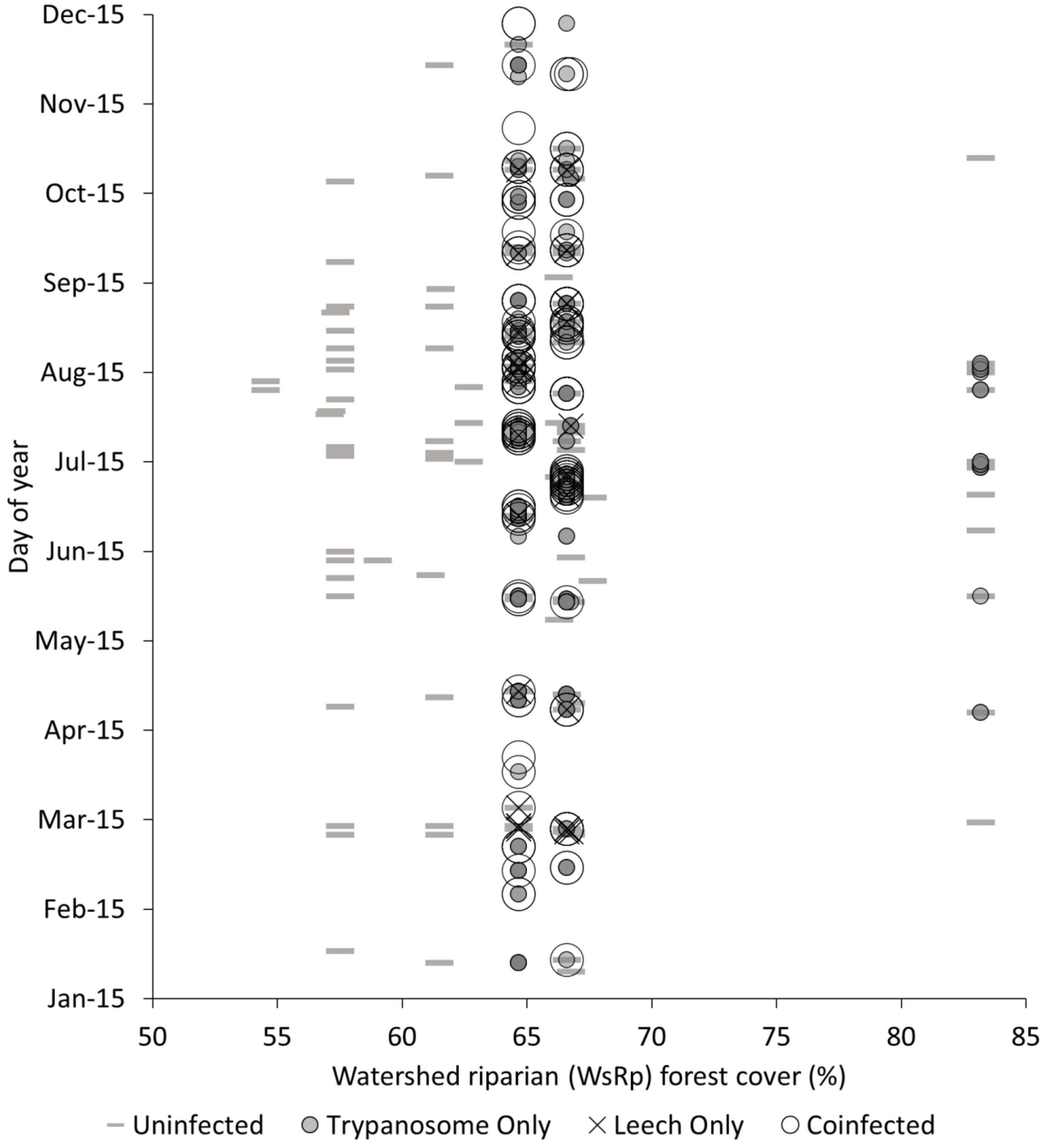

Supplemental Figure 2. Distribution of infection status among 841 hellbender (*Cryptobranchus alleganiensis*) capture events across a seasonal (y-axis) and land cover (x-axis) gradient.
